# Treatment of malignant sinonasal tumours with intensity-modulated radiotherapy (IMRT) and carbon ion boost (C12)

**DOI:** 10.1186/1471-2407-11-190

**Published:** 2011-05-22

**Authors:** Alexandra D Jensen, Anna V Nikoghosyan, Christine Windemuth-Kieselbach, Jürgen Debus, Marc W Münter

**Affiliations:** 1 Dept. of Radiation Oncology, INF 400, 69120 Heidelberg, Germany; 2 Alcedis GmbH, Winchester-Str. 2, 35394 Gießen, Germany

## Abstract

**Background:**

Most patients with cancers of the nasal cavity or paranasal sinuses are candidates of radiation therapy either due incomplete resection or technical inoperability. Local control in this disease is dose dependent but technically challenging due to close proximity of critical organs and accompanying toxicity. Modern techniques such as IMRT improve toxicity rates while local control remains unchanged. Raster-scanned carbon ion therapy with highly conformal dose distributions may allow higher doses at comparable or reduced side-effects.

**Methods/design:**

The IMRT-HIT-SNT trial is a prospective, mono-centric, phase II trial evaluating toxicity (primary endpoint: mucositis ≥ CTCAE°III) and efficacy (secondary endpoint: local control, disease-free and overall survival) in the combined treatment with IMRT and carbon ion boost in 30 patients with histologically proven (≥R1-resected or inoperable) adeno-/or squamous cell carcinoma of the nasal cavity or paransal sinuses. Patients receive 24 GyE carbon ions (8 fractions) and IMRT (50 Gy at 2.0 Gy/fraction).

**Discussion:**

The primary objective of IMRT-HIT-SNT is to evaluate toxicity and feasibility of the proposed treatment in sinonasal malignancies.

**Trial Registration:**

Clinical trial identifier NCT 01220752

## Background

Sinonasal malignancies are a heterogeneous group of tumours of the nasal cavity and paranasal sinuses accounting for about 5% of head and neck tumours and 1% of all cancers [1,2]. Most commonly, these are adenocarcinomas and squamous cell carcinomas, however also rare tumours such as adenoidcystic carcinomas, aesthesioneuroblastoma, sarcomas, melanomas, and other rare histologies are occasionally found in the nasal cavity and paranasal sinus [3]. Malignant sinonasal tumours are often asymptomatic until late in the course of the disease, therefore patients frequently present with extensive tumours displacing adjacent organs or infiltrating surrounding tissues. Due to the proximity of critical organs such as eyes, optic nerves, chiasm, lacrimal gland, temporal lobe, and pituary gland complete resection of these tumours is rarely possible. In addition, surgical en-bloc resections are impossible in this area of the body, which is another reason why surgical resection margins in locally advanced tumours are rarely free. Extensive surgical procedures are also severely mutilating if a radical maxillectomy or orbital exenteration are necessary to remove the tumour. Chemotherapy for the treatment of sinunasal malignancies is still under discussion, however, recent results showed a significant advantage for postoperative adjuvant chemotherapy either alone or in combination with radiotherapy [4].

In view of the rarely complete surgical therapy, most patients will undergo radiation therapy at one point in the course of their treatment. Maximal surgical resection followed by adjuvant conventional radiotherapy leads to a local control of 59% and an overall survival of 40% at 5 years [5]. Unfortunately, conventional radiotherapy has led to therapy-induced loss of vision in approximately one third of the treated patients [6,7]. Recent years have seen the development of more sophisticated radiotherapy techniques. 3D conformal and intensity-modulated (IMRT) radiotherapy allow more conformal dose distributions and hence improved normal tissue sparing. Various planning studies were already able to demonstrate that especially patients with sinunasal tumours highly profit from modern RT-techniques [8-13]. Chen et al performed a retrospective analysis over five decades in their institution and could impressively show that also in practice introduction of new radiotherapy treatment techniques led to reduction of accompanying side-effects [14,15].

Recent clinical data though are showing promising results. Hoppe et al treated 37 patients with sinunasal malignancies with postoperative IMRT to a median dose of 60 Gy [16]. This treatment resulted in 2-year progression-free survival of 75% and an overall survival of 80%. At a median follow-up of 28 months, no grade 3/4 toxicity of the eyes or visual pathway was seen [16]. Another trial included 84 patients with various histologies of the paranasal sinuses also treating these patients with IMRT to a total dose of 70 Gy in 35 fractions either in the postoperative or definitive situation [3]. In this study, actuarial local control at 5 years was 70,7% with an overall survival of 67% at the time of analysis. Nineteen out of the 84 patients developed local recurrences of their tumour. Multivariate analysis was able to identify invasion of the cribriform plate and anterior cranial fossa as a significant predictor of lower local control [3,17]. Compared to Hoppe et al [16], dose escalation to 70 Gy in the Madani-series led to a higher rate of radiogenic early and late toxicity including increased rates of grade 2/3 eye-and optic nerve injury as well as high rates of grade 2/3 mucositis in this cohort [3]. In unresectable sinunasal tumours however, also Hoppe et al reported significant rates of acute and late toxicity [18]: two out of 39 patients developed radionecrosis while one patient suffered from unilateral loss of vision 7 years after RT with a dose of approximately 77 Gy to the optic nerve. However, the prognostic parameter for overall survival and local control were found to be T-stage [19] a total dose of >65 Gy [18]. Local failure within the high-dose area remains the predominant site of relapse [20,21] hence underlining the need for more aggressive treatment regimen. Therefore treatment concepts for these tumours remain a challenge: radiation oncologists and patients are confronted with the choice of either applying high radiation doses at the cost of significant side-effects or keeping side-effects low and risking higher rates of local relapse. In this situation though, particle therapy with highly conformal dose distributions and increased biological effectiveness might be a way out of the dilemma and increase therapeutic range. Initial steps have been made with a phase I/II trial using combined photon-/proton therapy [22]. Locally advanced paransal sinus tumours received a median total dose of 73.6 Gy yielding an actuarial local control of 82% at 5 years and an overall survival of 58% in this cohort [22]. Updated results of a larger patient cohort even showed 2-year local control rates of 86% at the cost of 25% grade III mucositis and 10% grade 2 ocular late toxicity [17].

Mucositis ≥ CTCAE°III is a very painful radiogenic acute toxicity leading to significant reduction of the patients' quality of life. In addition, higher grade mucositis may also lead to increased hospital admission for intensive supportive therapy including cost-intensive parenteral feeding. In addition, severe mucositis also leads to an increased number of therapy interruptions or even discontinuation of therapy and hence worsening o therapeutic outcome. In cases where additional chemotherapy is applied concomitantly, it is even more important to keep radiation-induced mucositis to a minimum to maintain compliance with the treatment regimen.

Both carbon ion therapy and IMRT have the potential to reduce normal tissue injury while allowing dose escalation within the tumour or prior tumour bed. The present trial (IMRT-HIT-SNT) was therefore designed to evaluate toxicity with special focus on mucositis ≥ CTCAE°III and efficacy in combined intensity-modulated RT and carbon ion boost.

## Methods/design

### Study design

The IMRT-HIT-SNT trial is a prospective, non-randomized phase II feasibility trial evaluating acute mucositis ≥ CTCAE°III as the primary endpoint.

### Study Characteristics

Based on the fact that local control seems to be dose-dependent and local failure within the high-dose area remains the predominant site of relapse [20,21], the combination of IMRT (50 Gy in 2 Gy/fraction) and C12-boost (24 GyE in 3 GyE/fraction) will be tested as to toxicity profile and efficacy.

### Study objectives

To evaluate feasibility and toxicity (with a focus on mucositis CTCAE °III) and efficacy of the treatment.

Incidence of mucositis ≥ CTC°III will be assessed as the primary endpoint of the trial, local control, disease-free survival, overall survival, toxicity (incl. mucositis CTCAE °I-II and late toxicity at 2 years post RT).

### Sample size/number of subjects

Incidence of mucositis CTC°III in the reported data was 14.1% for IMRT to similar total doses [3]. As described before, IMRT and carbon ion therapy promise a reduction of mucositis rates as compared to previous reports. We assume the new therapy to be clinically irrelevant if the incidence of mucositis ≥CTCAE°III is higher than 30% whereas the experimental therapy is clinically feasible and warranting further investigation if the rate of mucositis ≥CTCAE°III is less than 10% and hence more than 90% of patients do not experience a mucositis ≥CTCAE°III. Using Simons Two-stage-Design (α = 0.05; Power 80%) a sample size of 30 patients was caluclated assuming a drop out rate of 10%. In the first stage, a maximum of 2 out of 6 patients may show a mucositis ≥CTCAE°III, and 5 out of 27 patients in total [23].

### Patient selection

#### Inclusion criteria

• Histologically confirmed or incompletely resected adenocarcinoma or squamous cell carcinoma of the nasal cavity or paranasal sinuses

• Inoperable tumour or refusal to undergo surgical resection

• Macroscopic or microscopic residual tumour (R2/R1) or

• ≥T3/T4 or

• written informed consent

• pts aged 18 - 80 years

• effective contraception for pts in childbearing age (<12 months post beginning of menopause)

#### Exclusion criteria

• Prior radio- or chemotherapy for tumours of the head and neck

• Other previous malignancy within the past 5 years except prior, adequately treated basal cell carcinoma of the skin or pre-invasive carcinoma of the cervix

• Significant neurological or psychiatric condition including dementia or seizures or other serious medical condition prohibiting the patient's participation in the trial by judgement of the investigators

• Legal incapacity or limited legal capacity

• Positive serum/urine β-HCG/pregnancy

• Drug abuse

### Radiotherapy

#### Immobilisation/planning examinations

Patients are immobilized using individual thermoplastic head masks with thermoplastic shoulder fixation. Planning examinations consist of a planning CT scan (3 mm slice thickness) with the patient positioned in the individual fixation device and contrast-enhanced MRI for 3D image correlation.

#### Target volumes/dose prescription

CTV1 (carbon ion boost) includes the macroscopic tumour, positive lymph nodes and prior tumour bed with special focus on the R1-area. PTV1 consists of a 2 mm margin around the CTV1 but does not extend into critical organs at risk (i.e. brain stem, spinal cord).

We prescribe a dose of 24 GyE carbon ions in 3 GyE/fraction (5 fractions per week) to the CTV1, we aim at covering the CTV1 with the 95% prescription isodose. CTV2 includes CTV1 with safety margins along typical pathways of spread as well as the complete surgical operational area. Locoregional nodal areas (levels II and III) are also included within the CTV2, in case of radiologically suspect lympüh nodes, further levels are included as indicated. The CTV2 also takes account for set-up variations, hence corresponds to the PTV2 (CTV2 = PTV2). 50 Gy IMRT (inversely planned step-and-shoot or tomotherapy technique) in 25 fractions (5 fractions per week) are prescribed to the CTV2 (coverage at least with the 90% prescription isodose) taking into account doses applied by daily image guidance with MV-cone-beam CT.

### Planning and RT treatment technique

Carbon ion therapy treatment planning is carried out using a dedicated treatment planning system (TPS) developed for and in co-operation with HIT(Heidelberg ion therapy centre). TPS offers the following functionalities also expected in conventional radiation therapy as well as methods for biological RT treatment optimization. As ion beams exhibit an increased biological effective dose depending on various factors, these need to be included within the planning algorithm. In addition, steering parameters for scanned ion beams need also be calculated by the TPS.

Carbon ion treatment is given at the HIT after inverse treatment planning in active beam application (raster-scanning method). A monoenergetic ion beam with a full-width/half-maximum (FWHM) of 5mm is extracted from the accelerator system (synchrotron) and magnetically deflected to subsequently scan all planned iso-energetic slices roughly corresponding to the tumour's radiological depth. Using this method almost any desired dose distribution can be created.

Intensity-modulated RT is planned at two commercially available planning systems: KonRad (Siemens OS) for step-and-shoot applications or Tomotherapy^®^. IMRT hence is delivered either at a 6 MV-linear accelerator (step-and-shoot technique) or at a 6 MV tomotherapy unit.

Daily image guidance for carbon ion therapy consists of orthogonal x-ray controls in treatment position, for IMRT image guidance consists of MV cone-beam CTs.

### Supportive therapy

Patients do not routinely receive prophylactic feeding tubes, however if they uncommonly experience significant weight loss we will of course offer feeding tube insertion or parenteral feeding.

### Treatment schedule/follow-up

**Figure 1:**
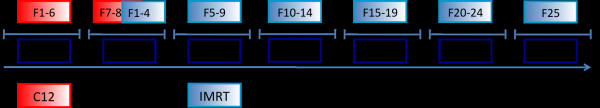
**Trial flow chart**.

After obtaining written informed consent, patients are included into the trial and receive their treatment planning investigations. Treatment starts with 8 fractions carbon ion (8 × 3 GyE C12) therapy followed by 27/28 fractions of IMRT corresponding to a total dose of approximately 74 GyE (figure 1). Treatment duration is approximately 6-7 weeks.

First follow-up examination including diagnostic, contrast-enhanced MRI will be carried out 6 weeks post completion of RT. Further controls including MRI are 3, 6, and 12 months thereafter, in 6 monthly intervals until 2 years post RT.

**Figure 2:**
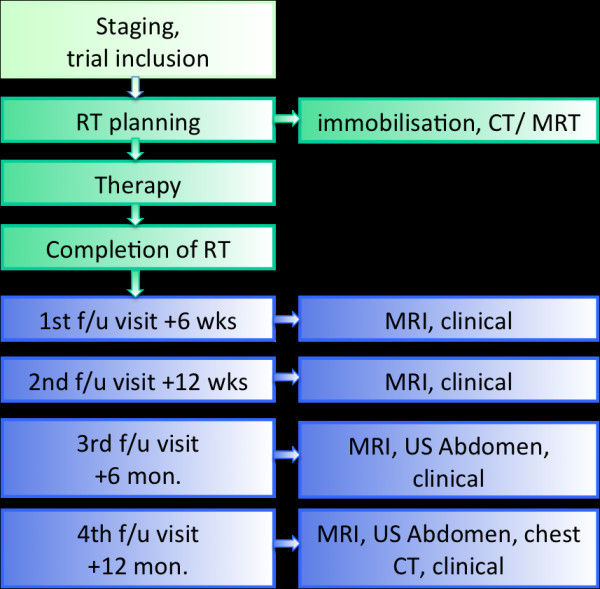
Follow-up.

Patients are also encouraged to undergo regular check-ups incl. full ENT clinical examinations and/or ophthalmological examinations as applicable in regular intervals (figure 2). Performance state (Karnofsky-Index), therapy-associated side effects as well as potential intercurrent therapy of each patient are recorded on follow-up.

### Proteomics and Genomics

For the proteomic examinations 30 mL venous blood will be collected from each subject prior to RT, at day 29, at completion of RT, and at the 1^st ^and 2^nd ^follow-up visit. Thus, the overall volume of blood samples used for Proteomic/Genomic investigations will be approximately 150 mL. Following parameters/pathways will be investigated:

• In order to predict the efficacy of the therapy blood will be collected during therapy and follow-up to detect and correlate the levels of well known tumour- and angiogenesis markers (VEGF, TGF-Alpha, bFGF, IL8, k-ras, etc.) using Enzyme-Linked Immunosorbent Assay (ELISA).

• Further, platelet protein content (i.e. tumor angiogenesis growth factors and cytokines) will be analyzed using citrate blood samples and correlated with serum- and plasma- protein results.

In order to perform the genomic analysis, patients' blood samples are collected as indicated and RNA, miRNA and DNA isolation will be performed. Based on an established platform, linear RNA-amplification, labelling and hybridization on human genome wide oligo-arrays (transcriptome analysis) are planned. DNA samples are used to identify potential chromosomal aberrations or epigenetic alterations that might predict treatment response. RNA and miRNA samples are further analyzed by real time quantitative RT-PCR to confirm microarray data and to test a subset of clinical predictors.

The determinations of proteomic and genomic parameters will be carried out at the Department of Radiation Oncology in Heidelberg. No further genetic investigations on the blood collected during the study will be carried out.

### Assessment of efficacy

Assessment of efficacy will be carried out by evaluation of imaging studies (MRI) at each follow-up. If applicable (in case of initial macroscopic tumour) tumour response will be evaluated according to the RECIST-criteria [24]. Occurrence of distant metastases (date and site) is recorded if applicable.

### Trial organization/coordination

The IMRT-HIT-SNT trial has been designed by the Department of Radiation Oncology, University of Heidelberg, and is carried out at the Heidelberg Ion Therapy Centre (HIT). It is an investigator-initiated trial; the Department of Radiation Oncology is responsible for co-ordination, overall trial management, registration (clinicaltrials.gov Identifier: NCT 01220752), database management, quality assurance, monitoring, and reporting.

### Investigators

Patients are recruited by the Department of Radiation Oncology.

### Adverse events

Adverse and serious adverse events are recorded using NCI common toxicity criteria for adverse events (CTCAE v.4). Acute radiation effects are defined as effects occurring within 90 days from beginning of radiotherapy. Late effects are defined as effects observed thereafter. Safety analysis is performed with respect to frequency of serious adverse events and adverse events stratified by organ system, severity, causality.

### Regular completion of the trial

Patient accrual is completed with inclusion of the last patient and should extend for approximately 4 years from trial initiation. Regular trial participation for each patient terminates 2 years post inclusion into the trial or the patient's death respectively.

### Discontinuation of treatment

• Patient wish

• Medical condition necessitating treatment termination and withdrawal of the patient from the trial

• Pregnancy

• Lack of compliance

### Premature termination of the trial

The trial can be prematurely closed or suspended by the LKP in following cases:

• Medical or ethical reasons relevantly affecting the risk-benefit relationship,

• Difficulties in recruitment of subjects suggest unjustifiable prolongation of the study timeline,

• Previously unexpected adverse events (in respect of their nature, severity, duration or outcome) occur with unjustifiable frequency,

• Expected adverse events occur with an unexpectedly high incidence,

• Relevant superiority of patients in one treatment arm of a comparable clinical trial,

• Legal authorities' decision

The Ethics Committee (EC) and the competent regulatory authorities will be informed about premature closure of the trial. Furthermore, the Ethics Committee(s) and competent regulatory authorities themselves may decide to stop or suspend the trial.

If the trial is closed prematurely, the trial material such as completed, partially completed, and blank CRFs will be returned to the coordinating investigator.

All involved investigators have to be informed immediately about a cessation or suspension of the trial. The decision is binding on all trial centers and investigators.

### Ethics, informed consent, and safety

The final protocol was reviewed by the ethics committee of the University of Heidelberg Medical School (S-319/2009). The trial complies with the Helsinki Declaration in its recent German version, the Medical Association's professional code of conduct, principles of Good Clinical Practice (GCP) guidelines and the Federal Data Protection Act. It will be carried out in keeping with local legal and regulatory requirements. It is also subject to authorization by the German radiation protection authority (Bundesamt für Strahlenschutz: = BfS). Medical confidentiality and Federal Data Protection Act will be followed. Written informed consent is obtained from each patient in oral and written form.

## Discussion

Sinonasal tumours are rare malignancies of the nasal cavity and paranasal sinuses. Preferred treatment would generally be complete surgical resection. Due anatomical site and proximity to critical structures surgical treatment is very complex and clear margins are rarely achieved. Therefore, the majority of patients will undergo RT either because of involved surgical resection margins or technical inoperability.

Outcome with adjuvant or definitive RT however has so far been hampered by high rates of accompanying toxicity. Modern radiotherapy techniques such as IMRT and image-guided RT (IGRT) have improved toxicity. Despite these sophisticated new techniques, it remains challenging to apply sufficient doses to the tumour in order to improve control rates.

This phase II trial was designed to evaluate the combination of 50 Gy IMRT plus 24 GyE carbon ion boost to a total dose of 74 GyE with respect to toxicity and control rates. To our knowledge, this is the first prospective trial evaluating this treatment regimen in sinonasal cancers.

## Competing interests

The authors declare that they have no competing interests.

## Authors' contributions

MWM, CWK, and JD developed the study protocol and planned the trial. CWK is responsible for statistical considerations/basis of the trial. ADJ, AN, MWM are responsible for conducting and co-ordination of the trial as well as patient recruitment. All authors read and approved the final manuscript.

## Pre-publication history

The pre-publication history for this paper can be accessed here:

http://www.biomedcentral.com/1471-2407/11/190/prepub
